# 1-Benzyl­idene-4-ethyl­thio­semicarbazide

**DOI:** 10.1107/S1600536810038444

**Published:** 2010-09-30

**Authors:** Yu-Feng Li, Yun-Cheng Zhang

**Affiliations:** a Microscale Science Institute, Department of Chemistry and Chemical Engineering, Weifang University, Weifang 261061, People’s Republic of China; b Department of Chemistry and Chemical Engineering, Weifang University, Weifang 261061, People’s Republic of China

## Abstract

The title compound, C_10_H_13_N_3_S, was prepared by the reaction of 4-ethyl­thio­semicarbazide and benzaldehyde. The dihedral angle between the benzene ring and the thio­urea unit is 8.96 (7)° and an intra­molecular N—H⋯N hydrogen bond generates an *S*(5) ring. In the crystal, inversion dimers linked by pairs of N—H⋯S hydrogen bonds generate *R*_2_^2^(8) loops.

## Related literature

For background to the coordination chemistry of Schiff bases, see: Habermehl *et al.* (2006 ▶). For a related structure, see: Li & Jian (2010 ▶).
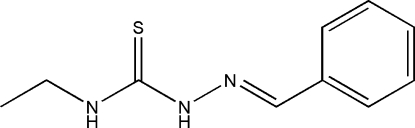


## Experimental

### 

#### Crystal data

C_10_H_13_N_3_S
*M* *_r_* = 207.30
Monoclinic, 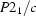

*a* = 8.4899 (17) Å
*b* = 13.467 (3) Å
*c* = 10.015 (2) Å
β = 96.04 (3)°
*V* = 1138.7 (4) Å^3^
*Z* = 4
Mo *K*α radiation
μ = 0.25 mm^−1^
*T* = 293 K
0.22 × 0.20 × 0.18 mm

#### Data collection

Bruker SMART CCD diffractometer
10048 measured reflections
2596 independent reflections
2118 reflections with *I* > 2σ(*I*)
*R*_int_ = 0.031

#### Refinement

*R*[*F* ^2^ > 2σ(*F* ^2^)] = 0.051
*wR*(*F* ^2^) = 0.152
*S* = 1.09
2596 reflections
127 parameters
H-atom parameters constrained
Δρ_max_ = 0.30 e Å^−3^
Δρ_min_ = −0.40 e Å^−3^

### 

Data collection: *SMART* (Bruker, 1997 ▶); cell refinement: *SAINT* (Bruker, 1997 ▶); data reduction: *SAINT*; program(s) used to solve structure: *SHELXS97* (Sheldrick, 2008 ▶); program(s) used to refine structure: *SHELXL97* (Sheldrick, 2008 ▶); molecular graphics: *SHELXTL* (Sheldrick, 2008 ▶); software used to prepare material for publication: *SHELXTL*.

## Supplementary Material

Crystal structure: contains datablocks global, I. DOI: 10.1107/S1600536810038444/hb5654sup1.cif

Structure factors: contains datablocks I. DOI: 10.1107/S1600536810038444/hb5654Isup2.hkl

Additional supplementary materials:  crystallographic information; 3D view; checkCIF report

## Figures and Tables

**Table 1:** Hydrogen-bond geometry (Å, °)

*D*—H⋯*A*	*D*—H	H⋯*A*	*D*⋯*A*	*D*—H⋯*A*
N1—H1*A*⋯N3	0.86	2.23	2.628 (2)	108
N2—H2*A*⋯S1^i^	0.86	2.74	3.5565 (16)	158

Symmetry code: (i) 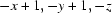.

**Fig. 1.**
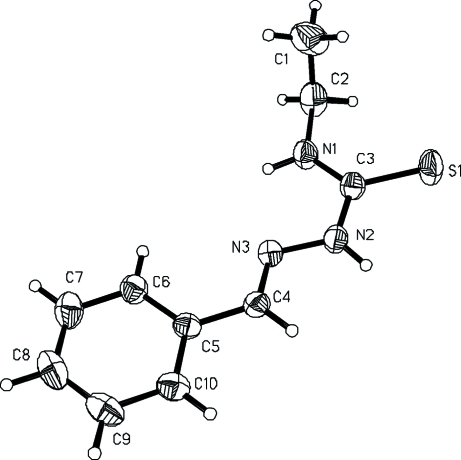
The structure of the title compound showing 30% probability displacement ellipsoids.

## supplementary crystallographic information

### Comment

Schiff bases are important intermediates which have been reported to be chiral coordination compound with many interesting properties (Habermehl *et al.*, 2006). As part of our research for new Schiff-base compounds we synthesized the title compound (I), and describe its structure here. In the molecule structure, the dihedral angle between the benzene ring and the thiourea unit is 8.96 (7)°.

Bond lengths and angles agree with those observed in a related structure (Li & Jian, 2010).

### Experimental

A mixture of 4-ethylthiosemicarbazide (0.1 mol) and benzaldehyde (0.1 mol) was stirred in refluxing ethanol (25 mL) for 2 h to afford the title compound (0.079 mol, yield 79%).  Colourless blocks of (I) were obtained by recrystallization from ethanol at room temperature.

### Refinement

H atoms were fixed geometrically and allowed to ride on their attached atoms, with C—H distances=0.97 Å, and with *U*_iso_=1.2–1.5*U*_eq_.

### Crystal data

**Table:**

C_10_H_13_N_3_S	*F*(000) = 440
*M**_r_* = 207.30	*D*_x_ = 1.209 Mg m^−^^3^
Monoclinic, *P*2_1_/*c*	Mo *K*α radiation, λ = 0.71073 Å
Hall symbol: -P 2ybc	Cell parameters from 2596 reflections
*a* = 8.4899 (17) Å	θ = 3.0–27.5°
*b* = 13.467 (3) Å	µ = 0.25 mm^−^^1^
*c* = 10.015 (2) Å	*T* = 293 K
β = 96.04 (3)°	Block, colorless
*V* = 1138.7 (4) Å^3^	0.22 × 0.20 × 0.18 mm
*Z* = 4	

### Data collection

**Table:**

Bruker SMART CCD diffractometer	2118 reflections with *I* > 2σ(*I*)
Radiation source: fine-focus sealed tube	*R*_int_ = 0.031
graphite	θ_max_ = 27.5°, θ_min_ = 3.0°
phi and ω scans	*h* = −10→9
10048 measured reflections	*k* = −17→17
2596 independent reflections	*l* = −13→13

### Refinement

**Table:**

Refinement on *F*^2^	Primary atom site location: structure-invariant direct methods
Least-squares matrix: full	Secondary atom site location: difference Fourier map
*R*[*F*^2^ > 2σ(*F*^2^)] = 0.051	Hydrogen site location: inferred from neighbouring sites
*wR*(*F*^2^) = 0.152	H-atom parameters constrained
*S* = 1.09	*w* = 1/[σ^2^(*F*_o_^2^) + (0.0834*P*)^2^ + 0.1728*P*] where *P* = (*F*_o_^2^ + 2*F*_c_^2^)/3
2596 reflections	(Δ/σ)_max_ < 0.001
127 parameters	Δρ_max_ = 0.30 e Å^−^^3^
0 restraints	Δρ_min_ = −0.40 e Å^−^^3^

### Special details

**Table:**

Geometry. All e.s.d.'s (except the e.s.d. in the dihedral angle between two l.s. planes) are estimated using the full covariance matrix. The cell e.s.d.'s are taken into account individually in the estimation of e.s.d.'s in distances, angles and torsion angles; correlations between e.s.d.'s in cell parameters are only used when they are defined by crystal symmetry. An approximate (isotropic) treatment of cell e.s.d.'s is used for estimating e.s.d.'s involving l.s. planes.
Refinement. Refinement of *F*^2^ against ALL reflections. The weighted *R*-factor *wR* and goodness of fit *S* are based on *F*^2^, conventional *R*-factors *R* are based on *F*, with *F* set to zero for negative *F*^2^. The threshold expression of *F*^2^ > σ(*F*^2^) is used only for calculating *R*-factors(gt) *etc*. and is not relevant to the choice of reflections for refinement. *R*-factors based on *F*^2^ are statistically about twice as large as those based on *F*, and *R*- factors based on ALL data will be even larger.

### Fractional atomic coordinates and isotropic or equivalent isotropic displacement parameters (Å^2^)

**Table:**

	*x*	*y*	*z*	*U*_iso_*/*U*_eq_	
S1	0.28967 (7)	0.39580 (4)	−0.07292 (4)	0.0744 (2)	
N1	0.25491 (17)	0.29999 (11)	0.15603 (14)	0.0596 (4)	
H1A	0.2916	0.2862	0.2372	0.072*	
N2	0.46620 (17)	0.40411 (10)	0.15771 (13)	0.0537 (3)	
H2A	0.5279	0.4416	0.1171	0.064*	
N3	0.50010 (15)	0.38467 (9)	0.29257 (13)	0.0479 (3)	
C4	0.61893 (18)	0.43036 (12)	0.35133 (16)	0.0504 (4)	
H4A	0.6768	0.4724	0.3014	0.061*	
C3	0.3357 (2)	0.36416 (12)	0.08914 (15)	0.0516 (4)	
C5	0.66728 (17)	0.41886 (11)	0.49435 (16)	0.0473 (3)	
C6	0.5847 (2)	0.35906 (13)	0.57650 (17)	0.0566 (4)	
H6A	0.4966	0.3235	0.5399	0.068*	
C10	0.7983 (2)	0.47106 (14)	0.55174 (19)	0.0624 (4)	
H10A	0.8542	0.5117	0.4983	0.075*	
C7	0.6330 (3)	0.35251 (16)	0.71139 (19)	0.0721 (5)	
H7A	0.5773	0.3125	0.7658	0.087*	
C9	0.8465 (2)	0.46324 (17)	0.6869 (2)	0.0765 (6)	
H9A	0.9356	0.4977	0.7238	0.092*	
C8	0.7637 (3)	0.40488 (16)	0.7670 (2)	0.0775 (6)	
H8A	0.7953	0.4005	0.8586	0.093*	
C1	−0.0363 (3)	0.3091 (2)	0.1253 (3)	0.1024 (9)	
H1B	−0.1290	0.2740	0.0876	0.154*	
H1C	−0.0324	0.3729	0.0832	0.154*	
H1D	−0.0405	0.3176	0.2200	0.154*	
C2	0.1083 (3)	0.25096 (16)	0.1020 (2)	0.0777 (6)	
H2B	0.1027	0.1862	0.1435	0.093*	
H2C	0.1102	0.2410	0.0063	0.093*	

### Atomic displacement parameters (Å^2^)

**Table:**

	*U*^11^	*U*^22^	*U*^33^	*U*^12^	*U*^13^	*U*^23^
S1	0.0973 (4)	0.0820 (4)	0.0422 (3)	−0.0240 (3)	−0.0006 (2)	0.00516 (19)
N1	0.0682 (9)	0.0602 (8)	0.0491 (7)	−0.0158 (7)	0.0001 (6)	0.0046 (6)
N2	0.0586 (8)	0.0584 (8)	0.0443 (7)	−0.0070 (6)	0.0066 (6)	0.0044 (5)
N3	0.0514 (7)	0.0472 (7)	0.0451 (7)	0.0009 (5)	0.0046 (5)	0.0014 (5)
C4	0.0470 (8)	0.0513 (8)	0.0534 (8)	−0.0022 (6)	0.0070 (6)	0.0066 (7)
C3	0.0598 (9)	0.0503 (8)	0.0450 (8)	−0.0013 (7)	0.0070 (6)	−0.0036 (6)
C5	0.0444 (7)	0.0436 (7)	0.0533 (8)	0.0039 (6)	0.0026 (6)	0.0001 (6)
C6	0.0599 (9)	0.0524 (8)	0.0572 (9)	−0.0016 (7)	0.0049 (7)	0.0063 (7)
C10	0.0529 (9)	0.0616 (10)	0.0714 (11)	−0.0050 (8)	0.0000 (8)	−0.0015 (8)
C7	0.0932 (14)	0.0654 (11)	0.0584 (10)	0.0106 (10)	0.0110 (9)	0.0107 (9)
C9	0.0693 (12)	0.0740 (12)	0.0806 (13)	0.0041 (10)	−0.0182 (10)	−0.0179 (10)
C8	0.0976 (15)	0.0781 (13)	0.0528 (10)	0.0246 (11)	−0.0108 (10)	−0.0087 (9)
C1	0.0765 (14)	0.0977 (18)	0.126 (2)	−0.0322 (14)	−0.0217 (14)	0.0169 (15)
C2	0.0950 (15)	0.0722 (12)	0.0629 (11)	−0.0365 (11)	−0.0059 (10)	0.0025 (9)

### Geometric parameters (Å, °)

**Table:**

S1—C3	1.6838 (16)	C10—C9	1.376 (3)
N1—C3	1.328 (2)	C10—H10A	0.9300
N1—C2	1.462 (2)	C7—C8	1.382 (3)
N1—H1A	0.8600	C7—H7A	0.9300
N2—C3	1.352 (2)	C9—C8	1.370 (3)
N2—N3	1.3761 (18)	C9—H9A	0.9300
N2—H2A	0.8600	C8—H8A	0.9300
N3—C4	1.272 (2)	C1—C2	1.495 (4)
C4—C5	1.456 (2)	C1—H1B	0.9600
C4—H4A	0.9300	C1—H1C	0.9600
C5—C10	1.389 (2)	C1—H1D	0.9600
C5—C6	1.392 (2)	C2—H2B	0.9700
C6—C7	1.373 (2)	C2—H2C	0.9700
C6—H6A	0.9300		
			
C3—N1—C2	124.92 (15)	C6—C7—C8	120.6 (2)
C3—N1—H1A	117.5	C6—C7—H7A	119.7
C2—N1—H1A	117.5	C8—C7—H7A	119.7
C3—N2—N3	119.89 (13)	C8—C9—C10	120.19 (19)
C3—N2—H2A	120.1	C8—C9—H9A	119.9
N3—N2—H2A	120.1	C10—C9—H9A	119.9
C4—N3—N2	115.84 (13)	C9—C8—C7	119.75 (19)
N3—C4—C5	122.10 (14)	C9—C8—H8A	120.1
N3—C4—H4A	118.9	C7—C8—H8A	120.1
C5—C4—H4A	118.9	C2—C1—H1B	109.5
N1—C3—N2	116.22 (14)	C2—C1—H1C	109.5
N1—C3—S1	124.84 (13)	H1B—C1—H1C	109.5
N2—C3—S1	118.92 (13)	C2—C1—H1D	109.5
C10—C5—C6	118.67 (15)	H1B—C1—H1D	109.5
C10—C5—C4	118.95 (15)	H1C—C1—H1D	109.5
C6—C5—C4	122.37 (14)	N1—C2—C1	112.78 (18)
C7—C6—C5	120.11 (17)	N1—C2—H2B	109.0
C7—C6—H6A	119.9	C1—C2—H2B	109.0
C5—C6—H6A	119.9	N1—C2—H2C	109.0
C9—C10—C5	120.71 (18)	C1—C2—H2C	109.0
C9—C10—H10A	119.6	H2B—C2—H2C	107.8
C5—C10—H10A	119.6		

### Hydrogen-bond geometry (Å, °)

**Table:**

*D*—H···*A*	*D*—H	H···*A*	*D*···*A*	*D*—H···*A*
N1—H1A···N3	0.86	2.23	2.628 (2)	108
N2—H2A···S1^i^	0.86	2.74	3.5565 (16)	158

Symmetry codes: (i) −*x*+1, −*y*+1, −*z*.
